# Rubberband Effect in Temporal Control of Mismatch Negativity

**DOI:** 10.3389/fpsyg.2016.01299

**Published:** 2016-08-31

**Authors:** Lingyan Wang, Xiaoxiong Lin, Bin Zhou, Ernst Pöppel, Yan Bao

**Affiliations:** ^1^School of Psychological and Cognitive Sciences, Key Laboratory of Machine Perception (Ministry of Education) and Beijing Key Laboratory of Behavior and Mental Health, Peking UniversityBeijing, China; ^2^Departments of Neurosurgery and Neuroscience, Baylor College of Medicine, HoustonTX, USA; ^3^Institute of Psychology, Chinese Academy of SciencesBeijing, China; ^4^Institute of Medical Psychology and Human Science Center, Ludwig-Maximilians-UniversityMunich, Germany

**Keywords:** event related potential, mismatch negativity, oddball paradigm, time window, correlation

## Abstract

Mismatch negativity (MMN) is a difference event-related potential (ERP) wave reflecting the brain’s automatic reaction to deviant sensory stimuli, and it has been proven to be a useful tool in research on cognitive functions or clinical disorders. In most MMN studies, amplitude, peak latency, or the integral of the responses, in rare cases also the slopes of the responses, have been employed as parameters of the ERP responses for quantitative analyses. However, little is known about correlations between these parameters. To better understand the relations between different ERP parameters, we extracted and correlated several different parameters characterizing the MMN waves. We found an unexpected correlation which gives new insight into the temporal control of MMN: response amplitudes are positively correlated with downside slopes, whereas barely correlated with upside slopes. This result suggests an efficient feedback mechanism for the MMN to return to the baseline within a predefined time window, contradicting an exponential decay function as one might expect. As a metaphor we suggest a rubberband effect for the MMN responses, i.e., the larger the distance of the response from neural equilibrium, the stronger the return force to equilibrium.

## Introduction

Mismatch negativity (MMN) is a negative event-related potential (ERP) component when subtracting brain responses to standard stimuli from those to rare stimuli, usually peaking at 150 to 250 ms after deviant onset (Näätänen et al., 2007). MMN was discovered by Näätänen et al. (1978) using an auditory oddball paradigm and equivalent responses have been observed in other sensory modalities such as in vision (Pazo-Alvarez et al., 2003) or in olfaction (Krauel et al., 1999). In a typical auditory oddball paradigm, an infrequent deviant sound is occasionally presented within a sequence of frequent standard sounds, and the participants either actively detect the deviant or ignore the entire sequence while focusing on signals of another modality. The elicitation of MMN in the latter case indicates that it can be observed independent of attention, and such independence is more pronounced in sleeping infants (Cheour et al., 2002; Martynova et al., 2003). MMN apparently discloses a neural mechanism to detect novel stimuli, and it is even elicited in complex situations when abstract rules are violated (Saarinen et al., 1992; Schröger et al., 2007).

In research on MMN, the amplitude of the response, usually quantified by the most negative value within the conventional MMN time window, has been proven to be a useful parameter (Sinkkonen and Tervaniemi, 2000; Paavilainen, 2013). Typically, the MMN amplitude gets larger when the oddball stimuli become more different, i.e., when the magnitude of oddball’s deviation from standard stimuli increases (Näätänen, 1992; Jaramillo et al., 2000; Pakarinen et al., 2007; but see Horváth et al., 2008). For example, Pakarinen et al. (2007) varied the frequency distance between the standard and deviant sounds and found that larger relative to smaller deviation (e.g., 523 and 609 Hz relative to 523 and 546 Hz) led to higher MMN amplitude measured at the Fz electrode. Similar relationships between the magnitude of deviation and the MMN amplitude can be demonstrated on other deviant dimensions as well, such as stimulus intensity (Pakarinen et al., 2007), duration (Jaramillo et al., 2000; Pakarinen et al., 2007), and perceived location (Pakarinen et al., 2007). Moreover, when the number of standard stimuli before a deviant increases, MMN amplitudes also get enhanced (Haenschel et al., 2005).

Beside amplitude, peak latency measured by the time from the deviant onset to the MMN peak is also widely used as an indicator in MMN research (Sinkkonen and Tervaniemi, 2000; Paavilainen, 2013). It has been observed that MMN peak latency gets shorter when stimuli deviation increases (Amenedo and Escera, 2000; Pakarinen et al., 2007). Both MMN amplitude and peak latency are good predictors of behavioral performances. While the accuracy of detecting deviants among series of standards is paralleled by the MMN amplitude (Lang et al., 1990, 1995; Jaramillo et al., 2000; Novitski et al., 2004; Pakarinen et al., 2007), the MMN peak latency in some cases predicts the speed of behavioral responses, i.e., the shorter the latency, the faster the reaction (Novitski et al., 2004; Pakarinen et al., 2007). Furthermore, both amplitude and latency have been well established as biomarkers in clinical cases of psychiatric disorders such as schizophrenia (Kargel et al., 2014) or autism (Roberts et al., 2011). These observations indicate that the MMN amplitude and latency are ecologically relevant indicators, which may reflect the neural operations of sensory novelty detection. Thus, an in-depth analysis of these indicators is necessary.

In previous ERP studies, amplitude or averaged amplitude in a short window are most frequently chosen for statistical analyses (Näätänen et al., 1978). In some cases temporal parameters like onset, offset or peak latency are also analyzed with the onset and offset latency being estimated by the time at the most positive value immediately before and after the MMN peak, respectively (Baldeweg et al., 1999). Sometimes the slopes of the waves indexed by the fluxion of the extrapolated line between the onset and the peak of MMN or the integral of the half MMN wave area are explored (Korostenskaja et al., 2003).

Although various parameters have been used in previous ERP studies, it remains unclear in detail how the different parameters are related to each other. Previous studies provide only limited evidence which shows no clear relationship between the MMN amplitude and different latencies (Lang et al., 1995). With respect to other ERP components rather than MMN, Polich et al. (1997) found negative correlations between the P3 amplitude and its latency, i.e., the larger the P3 amplitude, the shorter the latency, in a task requiring participants to discriminate an infrequent target from frequent standards; this correlation was somewhat biased to the right frontal electrodes, suggesting that the generation of P3 may involve initially the attentional control system of the right frontal cortex. In another study (Intriligator and Polich, 1994) it was found that the EEG power in lower frequency bands correlated positively with the P3 amplitude, suggesting a possible mediating role of attention resources. These observations indicate that exploring correlations between different parameters is a useful method to look into the neural mechanisms of ERP components.

The above-described research has examined mostly the relationship between the amplitude and latencies; other potentially indicative parameters such as upside and downside slopes are largely ignored. As suggested in some previous studies (Korostenskaja et al., 2003), the upside slope reflects the speed of the rise of MMN wave and indexes the speed of neuronal arousal processes associated with MMN responses. On this basis, it is important to extend the ERP analysis from focusing only on amplitude and latencies to more parameters such as upside and downside slopes as well as other temporal parameters.

**FIGURE 1:**
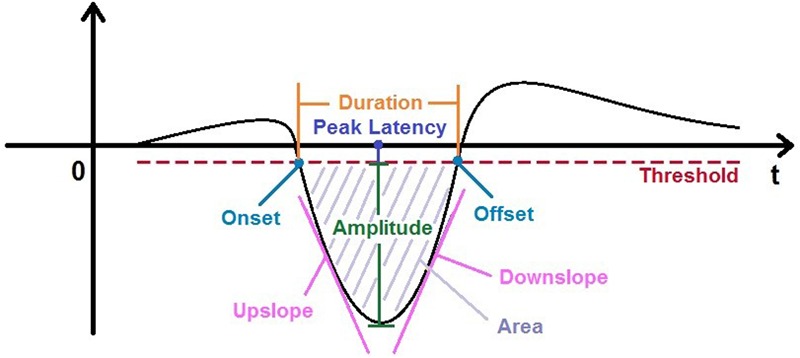
**Nine parameters characterizing the simplified mismatch negativity (MMN) wave**.

In the study presented here, we aimed to systematically examine nine parameters in the MMN waveform, i.e., onset, offset, peak amplitude, averaged peak amplitude (shortened as “ampavg”), duration, area, peak latency, upside and downside slopes (see **Figure 1**), trying to find out whether there exist certain correlations between different parameters of MMN. The data of this analysis were taken from a previous study (Wang et al., 2015), in which MMN amplitudes elicited by frequency deviants of sinusoidal tones in a passive oddball paradigm were compared for four inter-stimulus interval (ISI) conditions (1.5, 3, 4.5, and 6 s). The original results demonstrated significantly larger MMNs over central-frontal scalp areas for shorter ISIs up to 3 s as compared to longer ones, suggesting that the temporal modulation of 3 s provides a basic process of sequential segmentation which can be operated pre-attentively or pre-semantically (Pöppel, 1997, 2009). This finding also posed another question for the current analysis whether the suspected correlations could be present in all or a subset of ISI conditions. Considering MMN as a negative ERP component being present at various ISIs, we focused our analysis on the potential correlations between different MMN parameters, which might capture the substantial underlying neural processes of novelty detection. We anticipated that the suspected correlations should be independent of ISIs since their generation is due to generic operations of the neural system dealing with the departure from the equilibrium.

Among all the parameters we examined, latency, amplitude (including averaged amplitude) and slope (both upside slope and downside slope) were of special interest. Since previous study did not show any clear relationship between MMN latency and amplitude (Lang et al., 1995), we anticipated no correlation between MMN peak latency and amplitude or averaged amplitude as well. Since MMN slopes are waveform-related parameters while latency is a temporal one, we did not anticipate any correlation between MMN peak latency and slopes (both upside and downside slopes) as well. Regarding the relationship between MMN amplitude and slopes, which was of our major interest in the present study, we anticipated a different picture. As suggested previously (Korostenskaja et al., 2003), the upside slope indicates the speed of the rising MMN wave. Thus, the faster the rising speed, the larger the MMN amplitude. We hence hypothesized a positive correlation between MMN amplitude and upside slope. However, once the MMN wave reached its peak, neural attenuation processes were expected independent of the peak amplitude reflecting an exponential decay. Thus, we hypothesized no correlation between MMN amplitude and downside slope.

Taken together, our present study took a new perspective to gain better understanding of the neural processing underlying auditory change detection. We investigated systematically the relationships between different MMN parameters with certain predictions on the relationships between major parameters of our concern.

## Materials and Methods

### Participants

Twenty four right-handed Peking University students participated in this study; they received considerable financial reward afterward according to local standards. All participants passed an auditory test to guarantee normal hearing. They reported no neurological or psychiatric problems and had normal or corrected-to-normal vision. All the participants were informed that their brain activities would be recorded during the experiment, but they were naïve with respect to the real purpose of the study. The study was approved by the departmental ethics committee of Peking University, and all participants signed an informed consent before the experiment. In the data analysis, two participants were excluded from further analysis because of severe EEG artifacts; thus, 22 participants (11 females) remained for the final analyses. The mean age of the participants was 24.6 years (range 18–28 years).

### Stimuli and Procedure

Data were taken from a previous study, thus, the materials and methods, except the ERP data analysis, were identical to the study of Wang et al. (2015). A passive auditory oddball paradigm was employed in which a sinusoidal tone of 1000 Hz served as the standard stimulus and another sinusoidal tone of 1500 Hz served as the deviant stimulus. The occurrence probability of the deviant was 20%. Tones were of 100 ms duration and had an intensity of 65–75 dB when measured by a decibel meter at the ear locations. Video clips of a silent documentary movie about the Indian River Ganges served as task relevant stimuli to which participants were asked to pay attention during the experiment.

Auditory stimuli were presented with a constant ISI within each of eight blocks. Altogether four different ISIs (1.5, 3, 4.5, and 6 s) were used. They were assigned to the first four blocks in a Latin square order and to the remaining four blocks in a reversed order. Each block contained 150 auditory stimuli with 120 standard tones and 30 deviant tones. Deviant tones were separated from each other at least by two standard tones to avoid a potential decrease of the oddball effect. Furthermore, in each block the first five stimuli were standard tones, in order to set a baseline for the participants. During the experiment each participant sat in a comfortable armchair in a dimly lit and electrically shielded room. Participants were told to continuously watch the video clips while ignoring the auditory stimuli presented from a speaker 40 cm behind them. They were asked to pay full attention to the subtitles and video images, and were told that they would be tested during the breaks. About every 15 min participants were encouraged to take a break and they were quizzed by the experimenters on the movie contents. All participants memorized the movie content remarkably well. During the recordings, participants were asked to restrain from frequent eye blinks and head movements to avoid EEG artifacts. All experiments were done from 9 to 12 am to avoid potential circadian fluctuations (Pöppel and Bao, 2014; Zhou et al., 2014; Bao et al., 2015). On average, one recording lasted for about 90 min. After the experiment, participants were debriefed about the purpose of the experiment.

### Electrophysiological Recordings

Electroencephalographic (EEG) data were recorded with a 64-channel NeuroScan 4.3 system. The electrodes positions were chosen according to the extended 10–20 system. The average of the bilateral mastoids served as the online reference and the forehead served as ground. Vertical eye movements were monitored with bipolar electrodes above and below the left eye; horizontal eye movements were monitored with electrodes placed at the bilateral temples. The recording sampling rate was 500 Hz, high-pass filtered at 0.05 Hz and low-pass filtered at 100 Hz. In every recording, the impedance of each electrode was below 5 kΩ.

### EEG Data Preprocessing

Fieldtrip Toolbox (Oostenveld et al., 2011) was used for oﬄine pre-processing of EEG raw data. Raw data were first band-pass filtered between 1 and 100 Hz and then an independent component analysis (ICA, Belouchrani et al., 1993) was utilized to remove eye and muscle artifacts. We defined each epoch from 400 ms before to 800 ms after the onset of each stimulus. The ICA-processed data were corrected to the baseline of -200 ms to 0 ms and then low-pass filtered at 25 Hz. Deviant and standard epochs for each condition were separately averaged to obtain the waveforms. For each participant, standard waves were subtracted from deviant waves to obtain the MMN.

### ERP Parameters Extraction

In a first step, altogether nine categories were extracted from the MMN waves, i.e., amplitude, averaged amplitude, onset latency, offset latency, duration, area, peak latency, upside slope, and downside slope (named as upslope and downslope thereafter). Descriptions of each parameter and their defining criteria are listed in **Table 1** and shown in **Figure 1**. It should be mentioned that a threshold (Th) value was calculated in each MMN wave by the formula Th = Amp_base_-Std_base_, where Amp_base_ is the mean amplitude of the baseline epoch (from -200 ms to 0 ms) and the Std_base_ is the corresponding standard deviation. MMN waves without any amplitude more negative than Th were considered as “fake-MMNs” and discarded from further analysis.

**Table 1:** Parameters and their measuring criteria.

Parameter	Measuring criteria
Amplitude	Amplitude (in microvolts) of the most negative deflection (peak) between 100 and 300 ms after onset; for correlation analysis, its absolute value is used
Ampavg	Averaged value of ±20 ms around the peak; for correlation analysis, its absolute value is used
Onset	Time at the first point the MMN wave crossed the threshold
Offset	Time at the second point the MMN wave crossed the threshold
Peak latency	Time at the peak amplitude
Area	Accumulated area between onset and offset times
Duration	Offset minus onset time
Upslope	Largest derivative of the ascending side of the MMN wave; for correlation analysis, its absolute value is used
Downslope	Largest derivative of the descending side of the MMN wave; for correlation analysis, its absolute value is used

### Correlation Analysis

**FIGURE 2:**
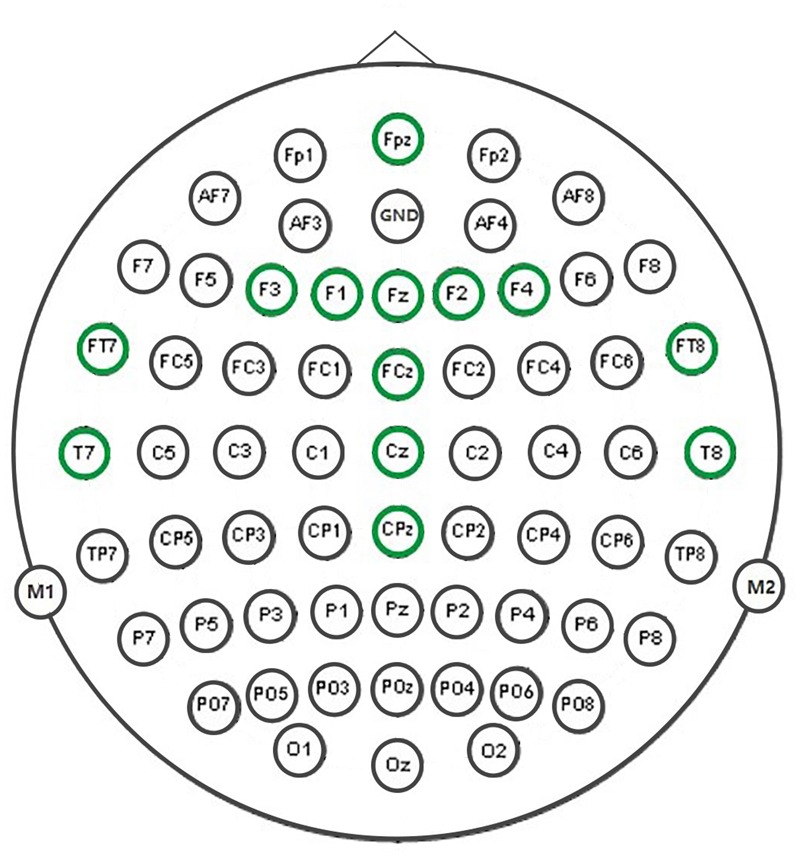
**Thirteen marked electrodes were used for the correlational analysis**.

Test of normality (Kolmogorov–Smirnov test) of parameters showed that some of them violated the normal distribution (ps > 0.05), and therefore Spearman Correlations (*n* = 22) coefficients were computed between all pairs of parameters for each of MMN waves, i.e., the waves for the four ISI conditions. Combinations of two out of nine parameters altogether result in $C_{9}^{2}$ = 36 pairs. Consequently, we have 36 × 4 = 144 correlation coefficients and corresponding *p*-values at each electrode. Thirteen electrodes were chosen for this correlation analysis, namely FPZ, FZ, FCZ, CZ, CPZ, F1, F3, F2, F4, FT7, FT8, T7, and T8, shown in **Figure 2**. These electrodes mainly are located over centro-frontal and temporal areas, and are often chosen as target electrodes for auditory MMN analysis (for a review see Näätänen et al., 2007). To this end, we first counted the number of significant and marginally significant correlations across electrodes for each ISI condition and each paired parameter. This approach does not simply test whether or not there is significant correlation among parameters, rather it calculates approximate indices for the relative reliability of the relationship between parameters based on the statistical tests, thus, reducing though not eliminating the potential impact of Type I error in drawing conclusions. More generally speaking, if one pair of parameters showed significant correlations at most of selected electrodes while another pair barely showed any, we would have decent confidence that the former (vs. the latter) revealed a relatively reliable relationship between the parameters. In this sense, although the temporal electrodes are close to the mastoids and exhibited smaller MMNs, the inclusion of them would not significantly alter the results and conclusions described below. Furthermore, ERP signals from adjacent electrodes might correlate with each other and our simple counting approach might conflate the strength of between-parameters relationship in some situations. Therefore, we conducted a further region of interest (ROI) analysis to first obtain averaged MMN waves within each of predefined regions and then calculate the parameters from the averaged MMNs for later correlation analysis. Altogether four regions were defined, i.e., frontal (FZ, FPZ, F1, F2, F3, and F4), central (FCZ, CZ, and CPZ), left temporal (T7 and FT7), and right temporal (T8 and FT8) regions.

## Results

On the basis of our analytical procedure we obtained a large number of correlation coefficients. To draw clear patterns from these correlations, we summed significant (*p* < 0.05; two-tailed and thereafter) or marginally significant (0.05 ≤ *p* < 0.1) correlations out of the 13 electrodes (**Figure 2**) for each condition (**Table 2**). For example, if seven electrodes out of 13 show significant or marginally significant correlations between two parameters, the corresponding number in **Table 2** would be 7. By counting the number of significant correlations across electrodes, we could obtain a first impression of the relative strength of the relationship between any two parameters.

**Table 2:** Counts of correlations across electrodes.

Pairs	1.5 s	3 s	4.5 s	6 s	Pairs	1.5 s	3 s	4.5 s	6 s
Ampavg-Amplitude	13 (13)	13 (13)	13 (13)	13 (13)	Upslope-Downslope	0	1 (1)	5 (2)	2 (2)
Ampavg-Upslope	1 (0)	1 (0)	0	1 (0)	Upslope-Duration	1 (1)	0	1 (0)	0
Ampavg-Peak latency	0	1 (0)	2 (0)	0	Upslope-Area	0	2 (0)	0	0
Ampavg-Onset	12 (9)	7 (4)	4 (1)	5 (3)	Peak latency-Onset	11 (10)	13 (13)	13 (13)	13 (13)
Ampavg-Offset	4 (2)	3 (2)	11 (7)	2 (2)	Peak latency-Offset	13 (13)	13 (13)	13 (13)	13 (13)
Ampavg-Downslope	8 (7)	7 (5)	7 (5)	8 (8)	Peak latency-Downslope	0	0	1 (0)	1 (0)
Ampavg-Duration	13 (13)	13 (13)	13 (13)	13 (12)	Peak latency-Duration	0	1 (0)	1 (0)	0
Ampavg-Area	13 (13)	13 (13)	13 (13)	13 (13)	Peak latency-Area	0	0	0	0
Amplitude-Upslope	0	1 (0)	1 (0)	0	Onset-Offset	5 (4)	10 (8)	9 (6)	13 (12)
Amplitude-Peak latency	0	0	0	0	Onset-Downslope	1 (1)	4 (2)	0	4 (1)
Amplitude-Onset	12 (10)	8 (5)	5 (3)	7 (5)	Onset-Duration	13 (13)	8 (6)	8 (8)	4 (3)
Amplitude-Offset	3 (2)	4 (2)	6 (6)	3 (3)	Onset-Area	13 (12)	8 (7)	7 (5)	6 (3)
Amplitude-Downslope	9 (9)	10 (9)	10 (7)	10 (10)	Offset-Downslope	0	1 (0)	0	0
Amplitude-Duration	13 (13)	13 (13)	13 (13)	13 (13)	Offset-Duration	9 (8)	11 (8)	12 (11)	11 (7)
Amplitude-Area	13 (13)	13 (13)	13 (13)	13 (13)	Offset-Area	8 (6)	9 (5)	12 (12)	6 (5)
Upslope-Peak latency	1 (0)	1 (0)	2 (2)	1 (1)	Downslope-Duration	1 (1)	0	2 (1)	3 (0)
Upslope-Onset	0	1 (0)	0	1 (1)	Downslope-Area	5 (3)	6 (3)	7 (4)	8 (6)
Upslope-Offset	1 (1)	2 (0)	2 (1)	1 (0)	Duration-Area	13 (13)	13 (13)	13 (13)	13 (13)

*Unparenthesized values, *p* < 0.1; parenthesized values, *p* < 0.05.*

As shown in **Table 2**, large amounts of correlations were found for several expected correlational pairs across electrodes. The first group of these pairs is due to their similarity in nature, and the typical example is the Ampavg-Amplitude which showed average *r* > 0.9 at all 13 electrodes. The second group is due to their mathematical relationship, examples including Amplitude-Area, Ampavg-Area, Onset-Duration, Offset-Duration, Onset-Area, and Offset-Area; within these pairs, the value of one parameter is dependent on the other parameter. Third, the large amounts of correlations for Amplitude-Duration, Ampavg-Duration, Peak Latency-Onset, and Peak Latency-Offset are expected in a commonsense way. For the other combinations, a clear trend was that temporal parameters (onset, offset, peak latency) and shape parameters (amplitude, ampavg, upslope and downslope) were usually not correlated as judged from the numbers across electrodes, consistent with the findings from Lang et al. (1995).

**FIGURE 3:**
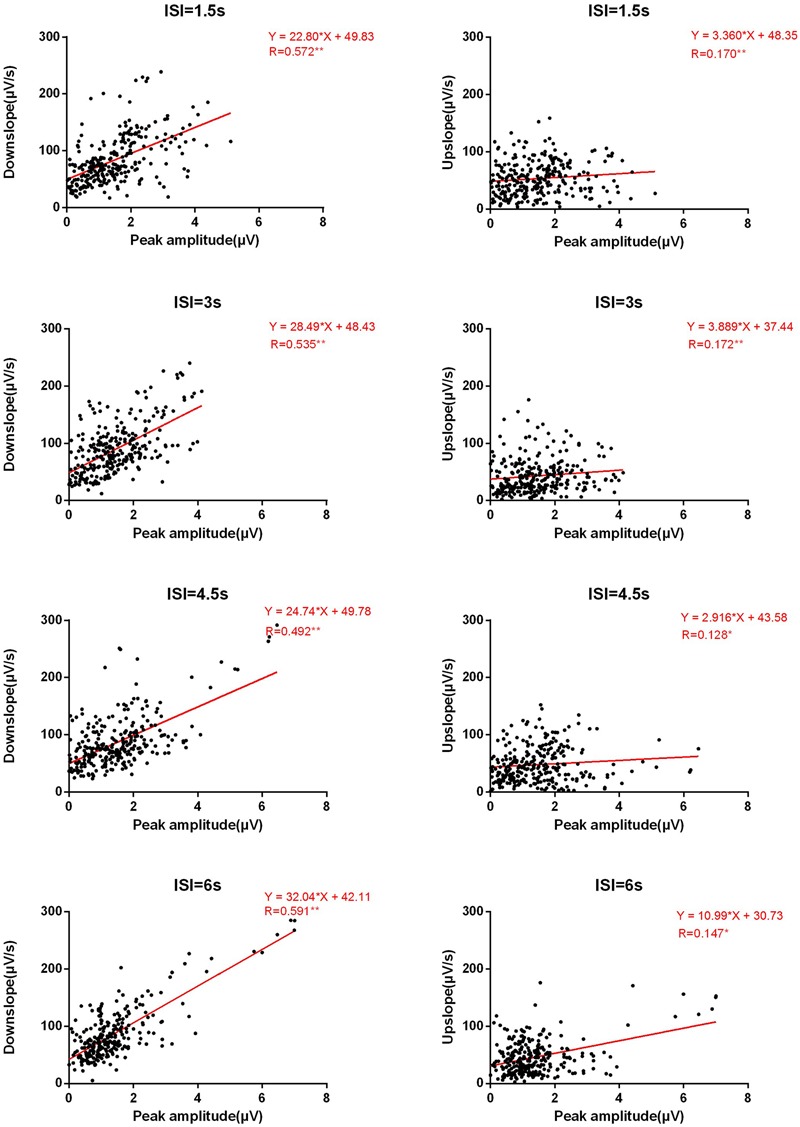
**Basic linear fitting of amplitudes and slopes.** The *Y*-axis represents the downslope **(left panels**) or the upslope **(right panels**) for four ISI conditions in absolute values. The *X*-axis represents the absolute values of peak amplitude. Each figure has the data points from all electrodes and participants.

Surprisingly, we found an unexpected asymmetry in correlational relationships between amplitude and upslope/downslope, which contradict our hypotheses. While rather high positive correlations in Amplitude-Downslope were observed (on average in more than 8 among 13 electrodes), the correlational relationships between Amplitude and Upslope barely exist (on average less than 1). A similar asymmetry was observed between Ampavg-Downslope (average number of correlated electrodes > 6) and Ampavg-Upslope (average number = 0). These correlations correspond to the low correlations between upslope and downslope (average number < 2). Furthermore, no systematic differences between the four ISI conditions were suspected from the counts in **Table 2**. To visually illustrate the results, in **Figure 3** for all electrodes and all ISIs the distribution of the two slopes (upslope and downslope) and the corresponding amplitudes are presented; a clear difference is seen between the Amplitude-Upslope and the Amplitude-Downslope relations.

Considering that ERP signals at nearby electrodes might correlate with each other, in a further step, we clustered the selected electrodes into four relatively homogenous regions and performed ROI analyses (see Materials and Methods). On the basis of the results in **Table 2**, we focused our ROI analyses on the correlations between amplitudes and slopes. The results are shown in **Table 3**. Consistent with the results of the counting analysis, **Table 3** shows significant correlations between Amplitude and Downslope but none between Amplitude and Upslope; the correlations between Ampavg with Downslope and Upslope showed equivalent results. Interestingly, the asymmetrical effects between Downslope and Upslope were more pronounced over the frontal and central scalp relative to the bilateral temporal areas.

**Table 3:** Correlations between amplitude and slopes across different regions.

Areas	Correlation Pairs	1.5 s	3 s	4.5 s	6 s
Frontal	Amplitude-Upslope	0.1553	0.3258	-0.0932	0.1218
	Amplitude-Downslope	0.6318^∗∗^	0.6533^∗∗^	0.7064^∗∗^	0.5504^∗^
Central	Amplitude-Upslope	0.1273	0.2532	0.1485	0.1182
	Amplitude-downslope	0.6805^∗∗^	0.2519	0.5675^∗∗^	0.6701^∗∗^
Left temporal	Amplitude-Upslope	0.3209	0.4180	-0.0597	-0.0120
	Amplitude-downslope	0.6017^∗∗^	0.5129^∗^	0.1779	0.0647
Right temporal	Amplitude-Upslope	-0.0536	0.0526	-0.3729	0.1818
	Amplitude-downslope	0.2208	-0.0180	0.3383	0.1195

*The table presents *R*-values of the correlations. ^∗^*p* < 0.05, ^∗∗^*p* < 0.01.*

## Discussion

Our study explored the potential correlations between parameters defining the time course and the shape of MMN wave. The MMNs were elicited using a conventional auditory oddball paradigm and responses in a group of 13 electrodes were analyzed. Consistent with our prediction, the MMN peak latency was not correlated with the amplitude (including ampavg), and no correlation was found for peak latency and MMN slope (upslope/downslope) either. In fact, our results showed that most combinations between temporal parameters (onset, offset, and peak latency) and wave shape parameters (amplitude, ampavg, upslope and downslope) were not correlated with each other, indicating that the MMN wave shape characteristics are irrelevant with respect to when the MMN is elicited.

**FIGURE 4:**
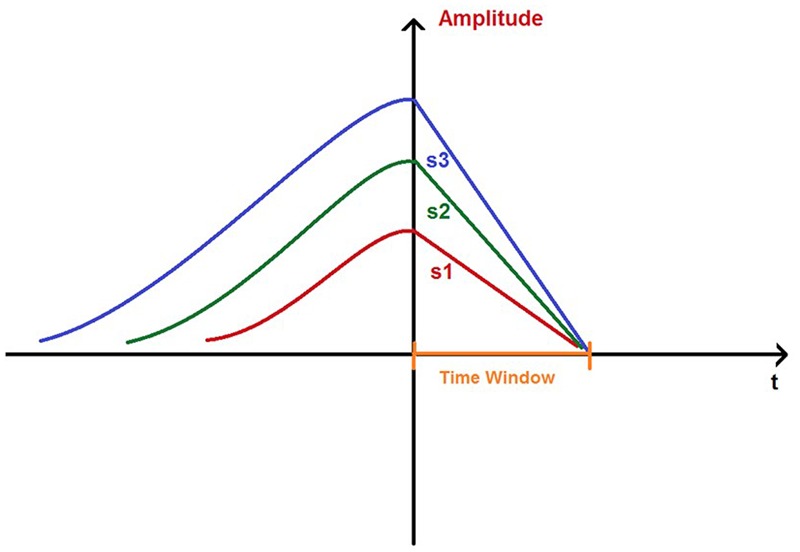
**A simplified sketch on a ‘time window’ for the MMNs to return to the baselines, ensured by slopes (s1, s2, and s3) that are correlated with amplitudes**.

Most importantly, we found an unexpected asymmetry regarding the relationship between MMN amplitude and the two types of slopes: the MMN downslope was positively correlated with the MMN amplitude, while the upslope was not correlated with the amplitude. This observation showed an opposite pattern to our hypothetical expectation and revealed an important new phenomenon: the downside decreasing speed of the MMN wave increases with the amplitude. To the best of our knowledge, it is the first time to observe such an asymmetric correlation between MMN amplitude and upslope vs. downslope. The observation that the MMN amplitude is positively correlated with the downslope and not with the upslope is very surprising, since an exponential decay as represented in many passively decreasing biological processes would be expected, i.e., once the MMN wave reaches its peak, the same neural attenuation processes should occur independent of the peak amplitude. The unexpected observation disproves our hypothesis and in our view suggests a different type of neural attenuation. Once the neural response reaches its peak, our brain uses a negative feedback mechanism to actively draw the neural activity back to the optimal operation level which can be referred to as the baseline; the more the deviation from the baseline, the stronger the return force to the baseline within a certain time (see **Figure 4**). This implies that apparently our neural system has a kind of “distance” information available (i.e., how far it is away from the baseline) at the time when the peak amplitude is reached, and can further use this information to actively control the returning process; otherwise, one could not explain that the returning speed is positively correlated with the distance from the baseline. This further suggests an anticipatory temporal control mechanism for the system to be prepared as fast as possible for the next novelty detection. Since the decreasing speed of MMN component is dependent on its deviation from the baseline, we suggest the name ‘rubberband effect’ as a metaphor to describe this mechanism. It is important to point out, that the effects were clearly observable in all ISIs which in a previous study (Wang et al., 2015) showed different MMN amplitudes. Thus, it seems that the association between the MMN amplitude and downslope might reflect an intrinsic neural mechanism dealing with the disequilibrium, which might be independent of the temporal segmentation (Pöppel, 1997, 2009) that modulates the MMN amplitude.

However, one has to be cautious about the generalization of this independence of the temporal segmentation. The dominant effect derived from the pattern of correlation coefficients (see **Table 3**) is observed in frontal midline areas extending back to central areas. Most importantly, however, we observed a clear hemispheric asymmetry. The right hemisphere did not show any involvement for a fast return to baseline; thus, there was no rubberband effect. However, in the left hemisphere the rubberband effect was observed for 1.5 and 3 s, but not for the longer ISIs. This observation suggests that in addition to the spatial distribution of the rubberband effect, there is also a temporal modulation of the effect. We speculate that the left hemisphere involvement of the effect and its temporal limitation to approximately 3 s could be related to the 3 s platform observed in verbal behavior; spontaneous speech is segmented in successive 3 s time windows (Vollrath et al., 1992). Thus, the brain is prepared for the next utterance within regular time intervals.

Contrary to our hypothesis as well, the MMN upslope was not correlated with the amplitude. This observation indicates that the generating (up) and diminishing (down) processes of MMN wave are implemented by different neural mechanisms. As indicated by some investigators, the rising phase may reflect the neuronal activation (Korostenskaja et al., 2003) or summation process (Zhou et al., 2010) during the encoding stage of information processing. The present study shows that this activation or summation process seems to be an integration process, which does not anticipate the position of the MMN peak. In other words, the MMN peak can be reached at any time during the summation process, thus resulting in no correlation between the upslope and the amplitude. The proposition that the MMN generating process does not anticipate the position of the peak is further supported by the observation of no correlation between the MMN amplitude and the peak latency. Thus, different from the rubberband-like active operation in the returning phase of the MMN, a passive information accumulation in the MMN generating process is indicated.

Besides our major surprising observation of an asymmetry of neural processes before and after the peak of the amplitude, we also observed that the MMN peak latency was not correlated with the MMN amplitude and the slopes, which is consistent with our hypotheses and also in line with observations by Lang et al. (1995). However, it should be noted that a previous study by Polich et al. (1997) did observe correlational relationships between amplitude and latency, but with the P3 ERP component. Since P3 is closely related to the operation of attention system (Polich, 2007) and the MMN in our case is observed with a passive oddball paradigm, our conclusion regarding the relationship between amplitude and latency should be limited to auditory frequency MMN in a passive oddball paradigm only, since the involvement of attention or the change of the oddball probability could possibly change the correlational relationships.

Finally, the current study also pointed out a simple yet effective data analyzing method by using correlational relationship as a way to explore psychological or neuronal mechanism in ERP signals. In previous studies, there have been discussions on advantages and disadvantages of single measurements like peak amplitude, peak latency or mean amplitude (Luck, 2014). Sometimes it may be difficult which parameter to use, as some might show hypothesized results while others do not. The method we suggest enables us to explore all parameters from a different perspective, and statistically the correlational analysis has a higher tolerance with respect to data variation, and it remains robust with different calculating methods. These advantages might prove to be useful in future studies.

## Author Contributions

LW, EP, and YB designed the experiment. LW, XL, and BZ collected and analyzed the data. LW, BZ, EP, and YB wrote the manuscript.

## Conflict of Interest Statement

The authors declare that the research was conducted in the absence of any commercial or financial relationships that could be construed as a potential conflict of interest.
